# Author Correction: Multi-layered cement-hydrogel composite with high toughness, low thermal conductivity, and self-healing capability

**DOI:** 10.1038/s41467-023-39431-3

**Published:** 2023-06-19

**Authors:** Yuan Chen, Yangzezhi Zheng, Yang Zhou, Wei Zhang, Weihuan Li, Wei She, Jiaping Liu, Changwen Miao

**Affiliations:** 1 Jiangsu Key Laboratory of Construction Materials, School of Materials Science and Engineering, Southeast University, Nanjing, 211189 China; 2 School of Transportation, Southeast University, Nanjing, 211189 China; 3 Jiangsu Key Laboratory of Advanced Metallic Materials, School of Materials Science and Engineering, Southeast University, Nanjing, 211189 China

**Keywords:** Bioinspired materials, Composites, Civil engineering, Mechanical properties

Correction to: *Nature Communications* 10.1038/s41467-023-39235-5, published online 10 June 2023

The original version of this Article contained a typographical error in the values of flexural toughness as described in the second paragraph of subsection ‘Structure and performance’ of the ‘Results’ section. The enhanced value of flexural toughness was incorrectly given as “14071 ± 153 kJ/m^3^” but should be “1407 ± 153 kJ/m^3^”.

Similarly, the original version of Figure 4a contained a typographical error in the legend of the pink line, which was incorrectly given as “W/C = 0.8 (Toughness = 1470 kJ/m^3^)” but should be “W/C = 0.8 (Toughness = 1407 kJ/m^3^)”.

This has been corrected in both the PDF and HTML versions of the Article.

