# Supplementary material for: Multi-layered cement-hydrogel composite with high toughness, low thermal conductivity, and self-healing capability
Source: Nat Commun. 2023 Jun 10;14:3438. doi: 10.1038/s41467-023-39235-5 (PMC10257691; doi:10.1038/s41467-023-39235-5)
Supplement: Supplementary file 1 — Supplementary Information [file 41467_2023_39235_MOESM1_ESM.pdf]

Supplementary Information

Multi-layered cement-hydrogel composite with high toughness, low thermal conductivity, and self-healing capability

Yuan Chen [a], Yangzezhi Zheng [b], Yang Zhou [a *], Wei Zhang [c *], Weihuan Li [a], Wei She [a], Jiaping Liu [a], Changwen Miao [a]

a Jiangsu Key Laboratory of Construction Materials, School of Materials Science and Engineering, Southeast University, Nanjing 211189, China
b School of Transportation, Southeast University, Nanjing 211189, China
c Jiangsu Key Laboratory of Advanced Metallic Materials, School of Materials Science and Engineering, Southeast University, Nanjing 211189, China
* corresponding author

Supplementary Figure

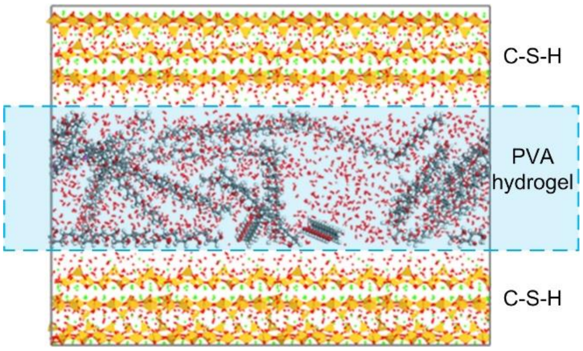

Supplementary Figure 1 A molecular dynamics simulation model for the interfacial interactions between a calcium silicate hydrate (C-S-H) matrix and polyvinyl alcohol (PVA) hydrogels in Materials Studio.

Supplementary Methods

The Clay Force Field (ClayFF) and Consistent Valence Force Field (CVFF) were employed to simulate the energy, structure, and dynamic properties of the C-S-H and PVA model, respectively.

In ClayFF force field, the interaction parameters between the different atoms are obtained according to the arithmetic mean rule for the distance parameter, R_0, and the geometric mean rule for the energy parameter, D_0:

$$R_{o,ij} = \frac{1}{2}\left(R_{0,i} + R_{0,j}\right) \tag{1}$$

$$D_{0,ij} = \sqrt{D_{0,i}D_{0,j}} \tag{2}$$

The short-range interactions are described by van der Waals energy represented via Lennard-Jones potential:

$$E_{VDW} = \sum_{i \neq j} D_{0,ij}\left[\left(\frac{R_{0,ij}}{r_{ij}}\right)^{12} - 2\left(\frac{R_{0,ij}}{r_{ij}}\right)^{6}\right] \tag{3}$$

The Coulombic potential is represented by:

$$E_{Coul} = \frac{e^2}{4\pi\epsilon_0}\sum_{i \neq j}\frac{q_i q_j}{r_{ij}} \tag{4}$$

where the partial charges q_i and q_j can be referred to Supplementary Table 1, e is the charge of the electron, and ϵ_0 is the dielectric permittivity of vacuum ($8.85419*10^{-12}$ F/m).

The bond stretch can angle bend in water molecules and hydroxyl groups can be described by simple harmonic potentials:

$$E_{bond\ stretch\ ij} = k_1\left(r_{ij} - r_0\right)^2 \tag{5}$$

$$E_{angle\ bend\ ijk} = k_2\left(\theta_{ijk} - \theta_0\right)^2 \tag{6}$$

where k_1 and k_2 are radial and angular stiffness, respectively. Parameters r_0 and θ_0 represent the equilibrium hydroxyl bond length and H-O-H angle in water molecules, respectively. (see Supplementary Table 2 and Table 3)

Supplementary Table 1 Non-bond Parameters for ClayFF Force Field

species	Symbol	Charge (e)	D_0 (kcal/mol)	R_0 (Å)
hydroxide calcium	cah	1.050	$5.0298*10^{-6}$	6.2428
aqueous calcium ion	Ca	2.000	0.1	3.2237
tetrahedral silicon	st	2.100	$1.8405*10^{-6}$	3.7064
bridging oxygen	ob	-1.050	0.1554	3.5532
hydroxyl oxygen	oh	-0.950	0.1554	3.5532
water oxygen	o*	-0.820	0.1554	3.5532
water hydrogen	h*	0.410		
hydroxyl hydrogen	ho	0.425		

Supplementary Table 2 Bond-stretch of water and hydroxyl groups for ClayFF Force Field

species i	species j	k_1 (kcal/mol/Å^2)	r_0 (Å)
h^*	o^*	554.1349	1.0000
ho	oh	554.1349	1.0000

Supplementary Table 3 Angle bend of water for ClayFF Force Field

species i	species j	species k	k_2 (kcal/mol/ θ^2)	θ_0
h^*	o^*	h^*	45.7696	109.47

In CVFF force field, non-bonded potential energy interactions E_{ij} between atoms i and j separated by a distance r were calculated according to:

$$E_{ij} = \frac{q_i q_j}{r} + 4\epsilon_{ij}\left[\left(\frac{\sigma_{ij}}{r}\right)^{12} - \left(\frac{\sigma_{ij}}{r}\right)^{6}\right] \tag{7}$$

where $\sigma_{ij} = \sqrt{\sigma_i \sigma_j}$ and $\epsilon_{ij} = \sqrt{\epsilon_i \epsilon_j}$ represent the van der Waals radius and energy well depth for the atomic pair. These parameters are given in Supplementary Table 4-7.

Supplementary Table 4 Non-bond Parameters for CVFF Force Field

species	Symbol	Charge (e)	D_0 (kJ/mol)	R_0 (Å)
methyl carbon	c3	-0.300	0.163	3.8754
sp^3 carbon bonded to 2H atoms	c2	-0.200	0.163	3.8754
sp^3 carbon bonded to 1H atom	c1	0.578	0.163	3.8754
hydrogen bonded to C	h	0.100	0.159	2.4500
hydrogen bonded to O	ho	0.350	0.000	0.0000
hydroxyl oxygen	oh	-0.380	0.650	3.1655

Supplementary Table 5 Bond-stretch for CVFF Force Field $E_{bond} = k_1(r - r_0)^2$

species i	species j	k_1 (kJ/mol/Å^2)	r_0 (Å)
c3	c2	1350.2	1.526
c3	h	1425.1	1.105
c2	oh	1606.7	1.420
c2	h	1425.1	1.105
oh	ho	2317.7	1.000

Supplementary Table 6 Angle bend for CVFF Force Field $E_{angle} = k_2(\theta - \theta_0)^2$

species i	species j	species k	k_2 (kJ/mol/rad ^{2})	θ_0(deg)
h	c3	c2	185.8	110.0
h	c3	h	165.3	106.4
c3	c2	oh	292.9	109.5
h	c2	c3	185.8	110.0
h	c2	oh	238.5	109.5
h	c2	h	165.3	106.4
c2	oh	ho	244.8	106.0
c2	c2	c2	195.0	110.5
c2	c2	h	185.8	110.0

Supplementary Table 7 Dihedral bend for CVFF Force Field $E_{dihedral} = k_3[1 + \cos(n\phi)]$

species i	species j	species k	species l	k_3 (KJ/mol)	d	n
h	c3	c2	oh	0.6615	1	3
h	c3	c2	h	0.6615	1	3
c3	c2	oh	ho	0.5439	1	3
h	c2	oh	ho	0.5439	1	3
c2	c2	c2	c2	0.6615	1	3
c2	c2	c2	h	0.6615	1	3
h	c2	c2	h	0.6615	1	3
