# Supplementary material for: Multi-layered cement-hydrogel composite with high toughness, low thermal conductivity, and self-healing capability
Source: Nat Commun. 2023 Jun 10;14:3438. doi: 10.1038/s41467-023-39235-5 (PMC10257691; doi:10.1038/s41467-023-39235-5)
Supplement: Supplementary file 3 — Description of Additional Supplementary Files [file 41467_2023_39235_MOESM3_ESM.pdf]

**Description of Additional Supplementary Files**

File Name: Supplementary Movie 1
Description: Includes the animation of the CSH-PVA interface during molecular dynamics simulations.

File Name: Supplementary Movie 2
Description: Includes the animation of the CSH-PVA model during a uniaxial tensile process of molecular dynamics simulations.

File Name: Supplementary Movie 3
Description: Includes the animation of crack propagation and fracture of cement-hydrogel under three-point bending stress in the FEM simulation.

File Name: Supplementary Movie 4
Description: Includes the animation of crack propagation and fracture of cement paste under three-point bending stress in the FEM simulation.
